# Fenofibrate and Lomitapide in *PNPLA2*-Related Neutral Lipid-Storage Disease with Myopathy: A Single-Patient In Vitro Approach

**DOI:** 10.3390/ijms27219609

**Published:** 2026-10-27

**Authors:** Kajus Merkevicius, Graeme Preston, Sadie Larson, Nathalie S. Houssin, Sander Houten, Tamas Kozicz, Eva Morava

**Affiliations:** 1 Department of Genetics and Genomic Sciences, Icahn School of Medicine at Mount Sinai, 1425 Madison Ave, New York, NY 10029, USA; 2 Clinic of Pediatrics, Institute of Clinical Medicine, Faculty of Medicine, Vilnius University, 03101 Vilnius, Lithuania; 3 Institute of Biosciences, Life Sciences Center, Vilnius University, 10257 Vilnius, Lithuania; 4 Department of Anatomy, University of Pecs Medical School, Pecs Szigeti út, 12 7624 Pecs, Hungary; 5 Department of Biophysics, University of Pecs Medical School, Pecs Szigeti út, 12 7624 Pecs, Hungary

**Keywords:** PNPLA2, neutral lipid-storage disease with myopathy, ATGL, fibroblasts, drug screening, Seahorse respirometry, lipid droplets, fenofibrate, lomitapide, personalized medicine

## Abstract

Neutral lipid-storage disease with myopathy (NLSD-M), caused by pathogenic *PNPLA2* variants, has no established treatment. We report a single-patient in vitro study in a 32-year-old female evaluating fenofibrate and lomitapide in patient-derived fibroblasts, selected based on prior therapeutic evidence. Therapeutic endpoints included lipid-droplet (LD) burden, quantified by volumetric z-stack imaging, and bioenergetic profiling, performed by Seahorse respirometry, citrate synthase (CS) activity, cellular NADH capacity, and complex II activity. Fibroblasts were treated with fenofibrate (20 µM) and lomitapide (5 nM) for two weeks. *PNPLA2*-mutant fibroblasts exhibited LD burden of median 56 droplets per cell, reduced CS activity, a proxy for mitochondrial content, and a hypometabolic profile. Preserved total NADH oxidase and Complex II activities indicated unaffected intrinsic electron transport chain function. Under fenofibrate exposure, most measured bioenergetic parameters in the patient-derived line were closer to the corresponding values in treated controls, but paradoxically increased LD count 1.5–1.9-fold. Lomitapide did not reduce LD burden and further worsened bioenergetic parameters. Loss of PNPLA2-mediated lipolysis produces a dual phenotype of LD accumulation and bioenergetic impairment. Fenofibrate and lomitapide produced divergent and partially adverse effects across these two endpoints. These findings informed precautionary, patient-specific clinical management decisions. Combined LD and metabolic profiling in patient-derived fibroblasts may complement therapeutic decision-making in NLSD-M, pending validation in larger cohorts.

## 1. Introduction

Neutral lipid-storage disorders (NLSDs) are rare autosomal recessive diseases characterized by intracellular non-lysosomal accumulation of triglyceride-containing lipid droplets (LD) [1]. Two main phenotypes have been described: neutral lipid-storage disorder with ichthyosis (NLSD-I, Chanarin-Dorfman syndrome; OMIM #275630) caused by pathogenic variants in *ABHD5*, and neutral lipid-storage disease with myopathy (NLSD-M; OMIM #610717), caused by pathogenic variants in *PNPLA2*. *ABHD5* has no known independent hydrolase activity; instead, it acts as a coactivator of PNPLA2 (also known as adipose triglyceride lipase, ATGL), the rate-limiting triglyceride lipase in adipose and other tissues [2]. Both conditions are characterized by lipid storage in skeletal and cardiac muscle, liver, bone marrow, peripheral leukocytes, and other tissues, with patients presenting with myopathy, cardiomyopathy, and other symptoms, and ichthyosis in NLSD-I [1,3,4]. Disease prevalence and the mechanisms linking impaired PNPLA2 function to clinical phenotype remain incompletely understood.

To date, more than one hundred *PNPLA2*-related NLSD-M cases have been reported worldwide and no disease-specific treatments have been established [1,4,5,6,7,8]. Limited preclinical and clinical evidence exists: (i) gene therapy in a *PNPLA2* knockout mouse model resolved lipid accumulation in cardiac muscle, with effects on skeletal muscle unreported [9,10]; (ii) screening of FDA-approved compounds in patient-derived *ABHD5*-related NLSD-I fibroblasts identified candidates affecting intracellular lipid content, for example LD lowering effect of lomitapide, which inhibits microsomal triglyceride transfer protein, reducing very-low-density lipoprotein assembly and circulating lipid availability [11]; (iii) fibrate (PPAR agonists) treatment in ATGL-deficient mice reversed mitochondrial defects, restored cardiac function, and prevented premature death [12] PPARα agonist; in human primary NLSD-M myotubes, ATGL deficiency caused intrinsic mitochondrial dysfunction accompanied by network fragmentation and reduced mitochondria–lipid-droplet contacts, and PPARδ agonism improved mitochondrial respiration in healthy but not ATGL-deficient cells, indicating that functional ATGL is required for PPAR agonism to exert its metabolic effects [13]; and (iv) Triheptanoin (Dojolvi^®^, Ultragenyx Pharmaceutical Inc., Novato, CA, USA) increased fractions of mitochondrial respiration and basal glycolysis in patient-derived fibroblasts from five NLSD-M patients [14]. A report described two NLSD-M patients treated with bezafibrate for 28 weeks, demonstrating decreased circulating triglyceride levels, increased metabolic flexibility, increased whole-body fat oxidation with fasting glucose oxidation normalizing to control levels, and decreased lipid accumulation in skeletal muscle, cardiac muscle, and liver [12]. However, these findings have not been independently replicated, and in the clinical setting treatment is based on individual patient-to-patient basis.

Loss of PNPLA2-mediated triglyceride lipolysis is associated with LD accumulation and altered cellular bioenergetics, providing complementary readouts for therapeutic evaluation. Previous studies established patient-derived fibroblasts as a model for therapeutic testing in NLSD-M, including lipid and bioenergetic assessment following triheptanoin exposure. Here, we extend this approach to fenofibrate and lomitapide, two compounds with prior preclinical or clinical rationale in NLSDs, using volumetric LD quantification and bioenergetic profiling in a single fibroblast line from a previously reported patient with *PNPLA2*-related NLSD-M. These exploratory in vitro findings informed a precautionary, patient-specific clinical management decision but do not establish treatment efficacy or safety.

## 2. Results

### 2.1. Baseline Phenotype of PNPLA2-Related NLSD-M Fibroblasts

#### 2.1.1. Lipid-Droplet Burden

**Figure 1:**
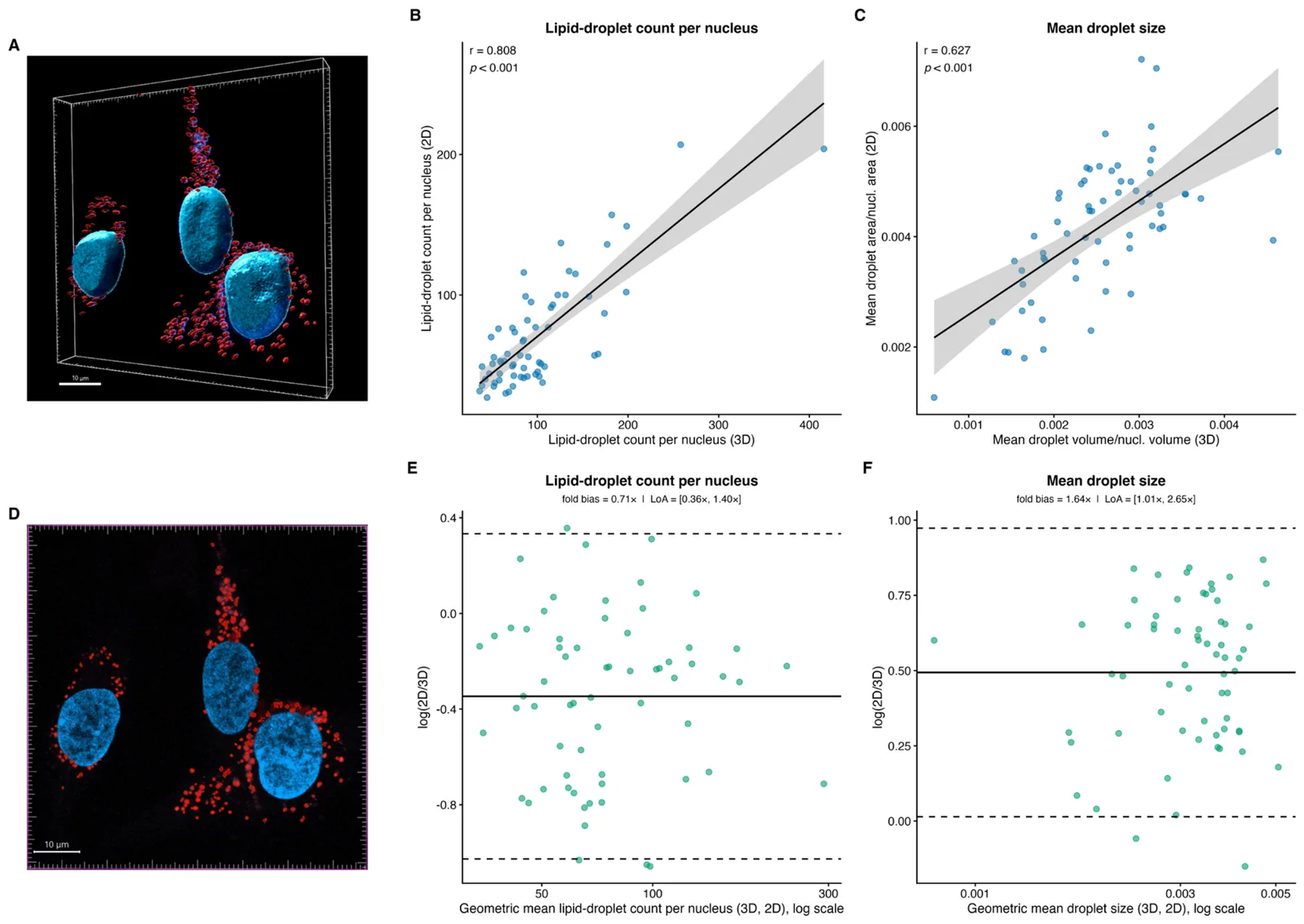
Comparison of microscopy methods for quantifying lipid-droplet burden in PNPLA2-mutant fibroblasts. (**A**) Representative 3D confocal reconstruction of Oil Red O-stained lipid droplets (red) and DAPI-stained nuclei (cyan); scale bar, 10 µm. (**B**,**C**) Correlation between matched 2D and 3D measurements of lipid-droplet count per nucleus (**B**) and mean droplet size (**C**), expressed as droplet area normalized to nuclear area (2D) or droplet volume normalized to nuclear volume (3D). Each point represents one field of view; black line shows linear regression fit with 95% confidence interval (grey band). Pearson r- and *p*-values are shown. (**D**) Representative single-plane image extracted computationally from the corresponding z-stack by selecting the z-position of the nucleus with centroid closest to the mean z-position of all nuclei in the field, approximating the equatorial plane of maximal nuclear cross-sectional area; scale bar, 10 µm. Extracted planes were segmented using identical parameters to the 3D analysis, enabling paired 2D/3D quantification on the same cells. (**E**,**F**) Bland–Altman plots comparing 2D and 3D quantification of lipid-droplet count per nucleus (**E**) and mean droplet size (**F**). The x-axis shows the geometric mean of paired measurements (log scale); the y-axis shows the log ratio of 2-D to 3-D values per field of view (green points). Solid line indicates mean fold bias; dashed lines indicate 95% limits of agreement.

In *PNPLA2*-mutant fibroblasts, LDs were visible on native phase contrast microscopy (Figure S1) as described in the literature [14,15,16], and quantified by Oil Red O (ORO)-stained 100× magnification (Figure 1). Untreated *PNPLA2*-mutant cells had a median of 56 (interquartile range, IQR 35–78) LD per cell, with mean droplet volume approximately 333- to 500-fold smaller than nuclear volume. Single-plane imaging systematically underestimated droplet count (fold bias 0.71×) and overestimated apparent droplet size (fold bias 1.64×) relative to volumetric quantification. Volumetric z-stack imaging was therefore used as the primary quantification method for all LD metrics.

#### 2.1.2. Bioenergetics Profile

**Figure 2:**
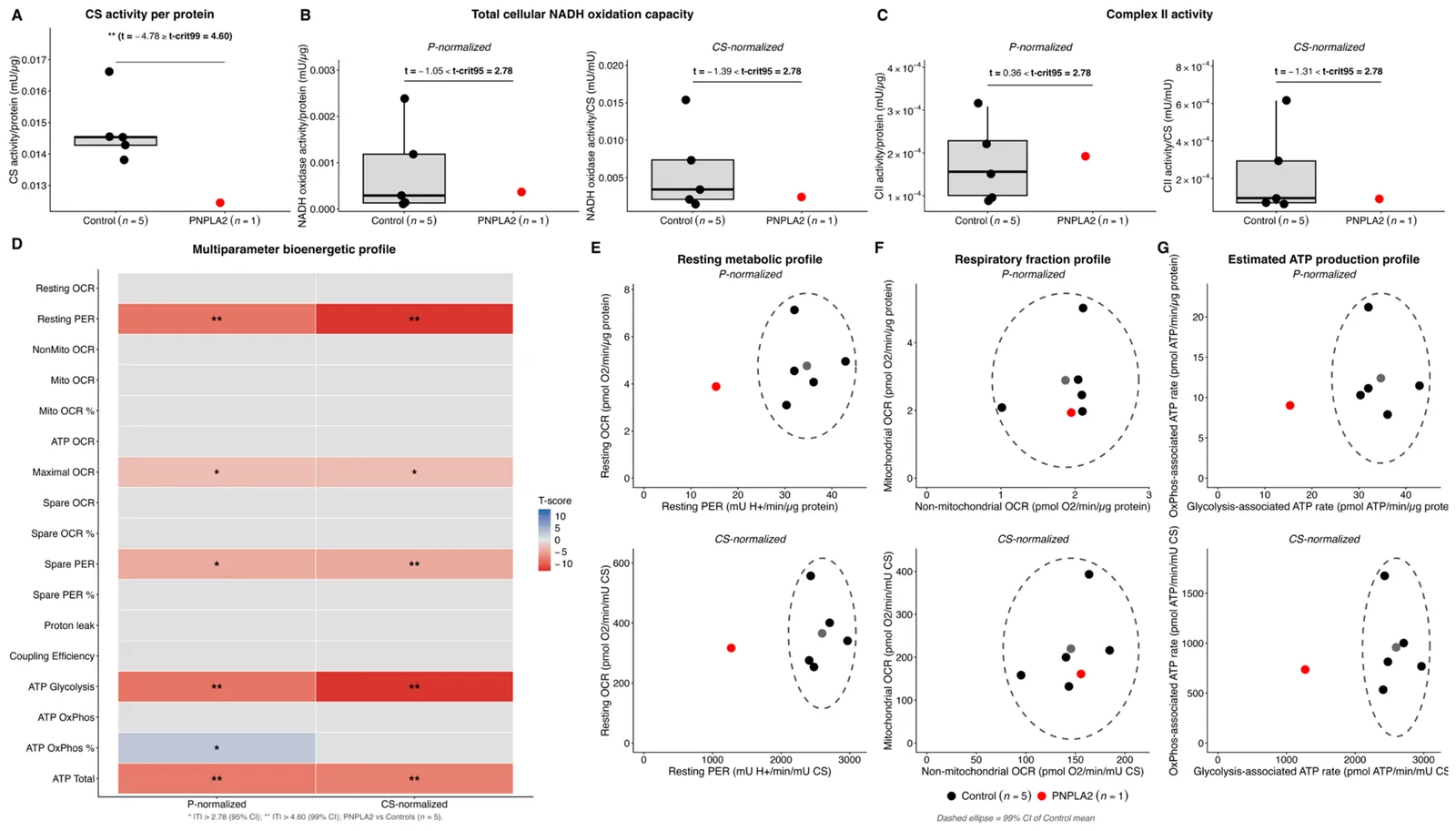
Baseline bioenergetic profile of *PNPLA2*-mutant fibroblasts compared to controls. (**A**) Citrate synthase (CS) activity normalized to total protein in control cell lines (black, *n* = 5) and the *PNPLA2*-mutant cell line (red, *n* = 1). (**B**) Total cellular NADH oxidation capacity and (**C**) Complex II activity, each shown normalized to total protein (P-normalized) and to CS activity (CS-normalized), in control (black, *n* = 5) and *PNPLA2*-mutant (red, *n* = 1) fibroblasts. As rotenone was not included in the NADH assay, panel B reflects total cellular NADH oxidation rather than isolated Complex I activity. Box plot shows median and interquartile range of controls; bracket reports T-score comparing *PNPLA2* to control mean (** = |T| beyond 99% confidence interval of the mean, CIM). (**D**) Multiparameter bioenergetic profile heatmap of T-scores for Seahorse-derived parameters comparing PNPLA2-mutant to control mean, shown for protein (P)- and citrate synthase (CS)-normalized data separately. Tile color indicates T-score direction and magnitude (blue = above controls, red = below controls); grey tiles indicate values within 95% CIM. Asterisks indicate significance thresholds: * = |T| > t-crit95; ** = |T| > t-crit99. Resting metabolic profile (**E**), respiratory fraction profile (**F**), and estimated ATP production profile (**G**) of *PNPLA2*-mutant fibroblasts (red) relative to controls (black, *n* = 5), shown for P-normalized (top) and CS-normalized (bottom) data. Axes show: resting proton efflux rate (PER) and resting oxygen-consumption rate (OCR) in (**E**); non-mitochondrial and mitochondrial OCR in (**F**); glycolytic and oxidative phosphorylation (OxPhos)-derived estimated ATP production rates in (**G**). Dashed ellipse indicates 99% CIM of the control group (grey point). All data represent untreated baseline conditions.

Compared to controls, NLSD-M fibroblasts showed reduced citrate synthase (CS) activity (outside 99% CIM, T = –4.78; Figure 2, a proxy for reduced mitochondrial content. *PNPLA2*-mutant fibroblasts exhibited a distinct phenotype (Figure 2 and Figure S2): (i) hypometabolic resting metabolic profile, driven by reduced resting proton efflux rate (PER), (ii) reduced maximal oxygen consumption rate (OCR), and (iii) reduced PER-derived glycolytic and OCR-derived total estimated ATP production rates (Table S1). Although maximal OCR fell outside the 95% CIM in the full cohort (T = −3.1) and within CIM boundaries in several leave-one-out subsets, T-scores remained consistently negative across all subsets and both normalization schemes (T-score range: −2.3 to −5.9), suggesting that this pattern may reflect a modest but consistent reduction rather than an artefact driven by any single control line (Figure S3). Total cellular NADH oxidation capacity and Complex II (CII) activity were both within the control range (Figure 2 and Figure S3), suggesting that intrinsic electron transport chain function may be preserved, and that the observed bioenergetic alterations could reflect reduced mitochondrial content, impaired substrate availability, or other upstream mechanisms rather than primary respiratory chain dysfunction.

#### 2.1.3. Autophagy Marker Abundance

LC3 B-II/LC3 B-I ratio, normalized to β-actin and used as a static indicator of autophagosome abundance under basal conditions [17], was similar between *PNPLA2*-mutant and control fibroblasts across three independent western-blot experiments (Figure S4), suggesting no detectable difference in basal autophagosome accumulation. As static LC3 B-II/LC3 B-I abundance does not measure autophagic flux, these findings do not exclude the possibility of altered flux due to compensatory changes in both autophagosome formation and clearance [18]. Therefore, static autophagy marker abundance was not used to assess treatment efficacy.

### 2.2. Treatment Effects in PNPLA2-Related NLSD-M Fibroblasts

**Figure 3:**
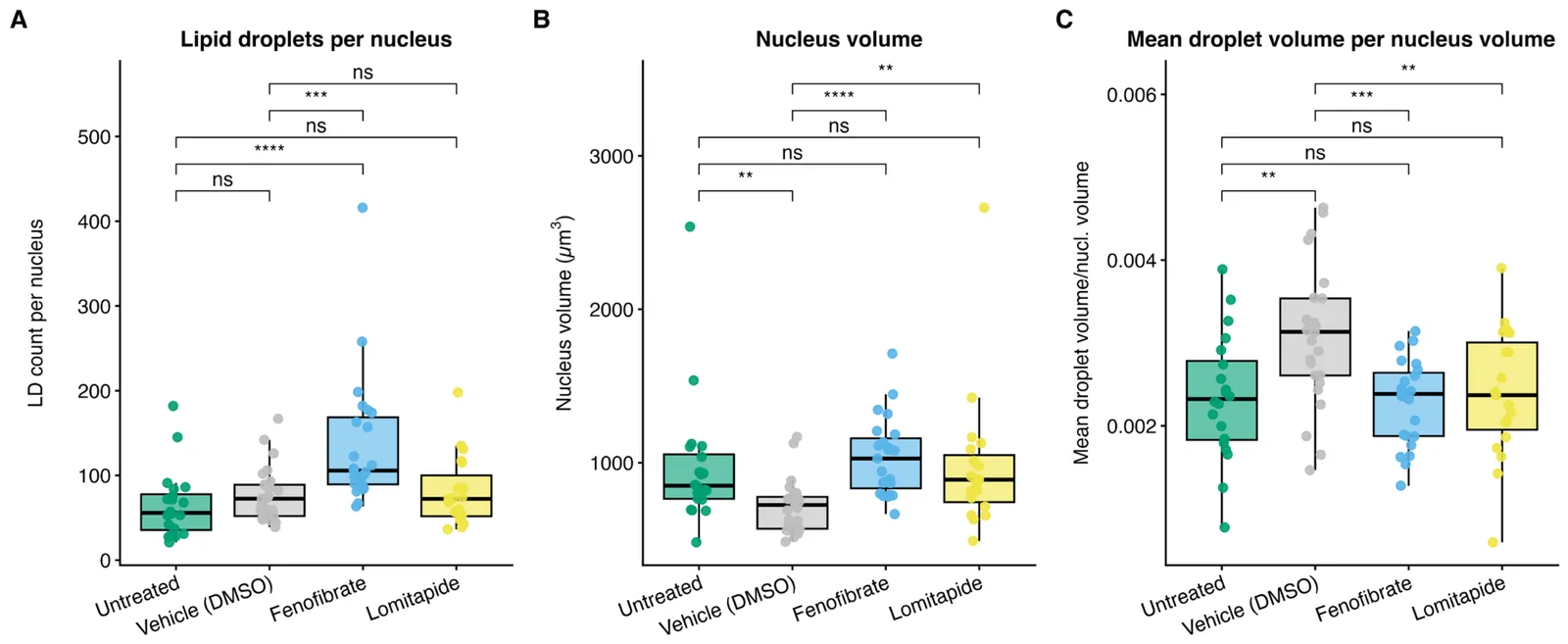
Lipid-droplet accumulation in PNPLA2-related NLSD-M patient fibroblasts across treatment conditions. Oil Red O-stained lipid droplets and DAPI-stained nuclei were quantified from volumetric z-stack images of *PNPLA2*-mutant fibroblasts under four conditions: untreated (green), vehicle (0.01% DMSO, grey), fenofibrate 20 µM (blue), and lomitapide 5 nM (yellow). Each condition represents a single biological culture of *PNPLA2*-mutant fibroblasts stained and imaged across four technical replicate wells. Each data point represents one field of view (1–4 cells per field); fields of view per condition: untreated *n* = 20, vehicle *n* = 25, fenofibrate *n* = 23, lomitapide *n* = 19. Boxes represent median with interquartile range (IQR); individual data points are shown. Parameters shown are lipid-droplet count per nucleus (**A**), nuclear volume (**B**), and mean droplet volume normalized to nuclear volume (**C**). Differences across conditions were assessed using Kruskal–Wallis tests followed by pairwise Wilcoxon rank-sum tests with Benjamini–Hochberg correction. Statistical comparisons reflect within-experiment field-of-view variability and should not be interpreted as evidence of a generalizable treatment effect in the absence of independent biological replicates. Brackets indicate comparison pairs (ns = not significant; ** *p* < 0.01; *** *p* < 0.001; **** *p* < 0.0001).

**Figure 4:**
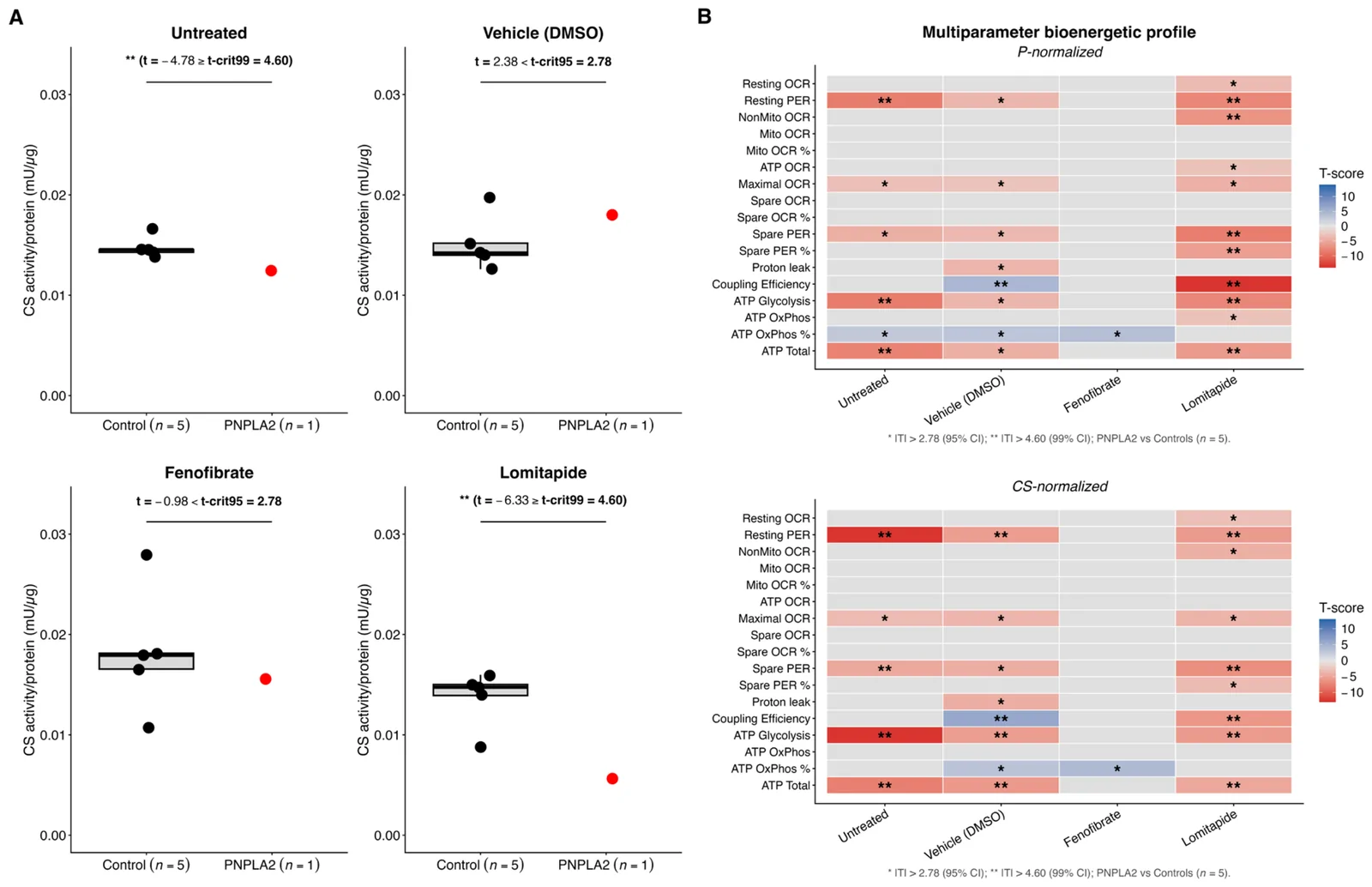
Effect of two-week fenofibrate and lomitapide treatment on citrate synthase activity and multiparameter bioenergetic profile in PNPLA2-mutant fibroblasts. (**A**) Citrate synthase (CS) activity normalized to total protein in control cell lines (black, *n* = 5) and the PNPLA2-mutant cell line (red, *n* = 1) across four treatment conditions: untreated, vehicle (0.01% DMSO), fenofibrate 20 µM, and lomitapide 5 nM. Brackets report the T-score and significance threshold for the PNPLA2-vs-control comparison within each condition (** = |T| beyond 99% CIM). (**B**) Multiparameter bioenergetic profile heatmaps of T-scores for Seahorse-derived parameters (rows) across treatment conditions (columns), comparing PNPLA2-mutant to control mean within each condition, shown separately for protein (P)-normalized (top) and CS-normalized (bottom) data. Tile color indicates T-score direction and magnitude (blue = above controls, red = below controls); grey tiles indicate values within 95% CIM. Asterisks indicate significance thresholds: * = |T| > t-crit95; ** = |T| > t-crit99.

Compared to untreated conditions, vehicle (0.01% DMSO) resulted in 1.3-fold increase in LD count and 1.2-fold reduction in nuclear volume (Figure 3). Beyond these LD burden changes, vehicle-treated *PNPLA2*-mutant cells had decreased proton leak with increased coupling efficiency and increased relative estimated OCR-derived OxPhos ATP production rate (Figure 4 and Figure S2). CS activity in *PNPLA2*-mutant fibroblasts, which was reduced relative to controls at baseline, converged to control levels under vehicle conditions. These findings are consistent with the capacity of low-dose DMSO to exert cell-type-dependent effects on mitochondrial bioenergetics [19]. All compound conditions were therefore compared to DMSO-treated cells to isolate drug-specific effects from vehicle background.

LD burden for each treatment condition was assessed within a single biological culture, the following comparisons reflect within-experiment field-of-view variability and are presented as exploratory. Fenofibrate treatment was associated with a median 1.9-fold increase in LD count per nucleus compared to untreated cells, and a 1.5-fold increase relative to vehicle-treated *PNPLA2*-mutant fibroblasts (Figure 3). Lomitapide did not affect LD count per nucleus beyond the vehicle effect alone (lomitapide vs. untreated: 1.3-fold; vehicle vs. untreated: 1.3-fold). Despite the increase in LD count, mean droplet volume per nucleus was 1.3-fold lower with fenofibrate relative to vehicle, consistent with the vehicle-associated reduction in nuclear volume rather than a compound-specific effect on droplet size; the same pattern was observed with lomitapide (1.3-fold lower vs. vehicle). Neither treatment produced a meaningful change in mean droplet volume per nucleus relative to untreated cells. These findings warrant confirmation in independent biological replicates.

After two weeks of lomitapide treatment, the metabolic profile of *PNPLA2*-mutant fibroblasts was further deviated from controls compared to vehicle alone, with additional decreases in: (i) non-mitochondrial OCR, (ii) resting and maximal OCR, coupling efficiency and estimated OCR-derived OxPhos ATP production rate (Figure 4 and Figure S5) relative to controls; these changes were not observed under vehicle conditions. Fenofibrate treatment resulted in a bioenergetic profile closely resembling that of controls across most parameters, with the exception of a relative increase in OCR-derived OxPhos ATP production rate—a change also observed in vehicle-treated cells. CS activity in *PNPLA2*-mutant cells was comparable to controls under both fenofibrate and vehicle conditions, in contrast to untreated and lomitapide-treated cells where CS activity was reduced (Figure 4).

Based on the negative effect on metabolic profile with no reduction in LD burden, lomitapide was not pursued clinically. Prior to in vitro testing, the 32-year-old NLSD-M patient had been treated with fenofibrate for six months without observed disease progression changes. Following the in vitro observation of increased LD accumulation, fenofibrate was discontinued as a precautionary, patient-specific clinical decision. As experiments were performed in fibroblasts without direct assessment of skeletal muscle lipid flux or fatty acid oxidation, this decision should not be interpreted as a causal conclusion.

## 3. Discussion

In this study, we evaluated two candidate compounds, fenofibrate and lomitapide, in a patient-derived fibroblast model from a 32-year-old female with *PNPLA2*-related NLSD-M using combined LD quantification and bioenergetic profiling. Building on established LD burden and hypometabolic bioenergetic profile in NLSD-M, we further demonstrate reduced CS activity, as proxy indicative of reduced mitochondrial content, with relatively preserved intrinsic electron transport chain (ETC) function—suggesting the metabolic phenotype may reflect reduced mitochondrial quantity, impaired substrate availability, or other upstream mechanisms rather than impaired ETC activity per organelle. Furthermore, pharmacological effects on lipid storage and cellular metabolism were not necessarily concordant. Under fenofibrate exposure, most measured bioenergetic parameters in the patient-derived line were closer to the corresponding values in treated controls while paradoxically increasing LD number. Whereas lomitapide did not reduce LD burden and in vitro bioenergetic profile had more parameters deviated from control range. These findings further support patient-derived fibroblasts as a platform for personalized therapeutic evaluation in NLSD-M, while demonstrating that LD burden alone may be insufficient to predict treatment response.

Fenofibrate treatment increased LD number without significantly altering mean droplet size, contrasting with a *FATP4* overexpression model in which LD size increased without a corresponding change in number [20]. Together, these observations suggest that distinct biological perturbations may produce different patterns of LD accumulation, potentially through enhanced de novo LD biogenesis versus expansion or fusion of existing droplets. LD number and size therefore provide non-redundant biological information.

Our findings corroborate previous findings of a distinct hypometabolic metabolic profile in NLSD-M [3,12,14,21]. Fatty acid oxidation (FAO) capacity has been reported as preserved in patient-derived fibroblasts but impaired in skeletal muscle biopsies, ATGL-deficient mice, and at the whole-body level in NLSD-M patients [3,12,21]. This study further expands the bioenergetic profile of NLSD-M by demonstrating reduced CS activity, as proxy indicative of reduced mitochondrial content, with PER- and OCR-derived parameter changes not proportional to CS activity reduction. Total cellular NADH oxidase capacity and CII activities were within the control reference range, indicating that intrinsic ETC function is preserved; the hypometabolic phenotype therefore more likely reflects reduced mitochondrial quantity, impaired substrate availability due to deficient lipolysis, or other upstream mechanisms, rather than impaired respiratory chain function per organelle. This pattern contrasts with findings in primary NLSD-M myotubes, where OxPhos complex protein content and mitochondrial DNA copy number were higher than in healthy controls despite markedly reduced basal, ATP-linked, and maximal respiration, indicating that in myotubes the bioenergetic deficit reflects intrinsic mitochondrial dysfunction rather than reduced quantity [13]. The divergence between dermal fibroblast and myotube findings likely reflects cell-type-specific differences in how ATGL deficiency manifests across tissues. In both cell types, however, the ATGL–PPAR–PGC-1α axis provides a plausible common mechanism: ATGL-mediated lipolysis generates lipid ligands for PPARα and PPARδ, which co-activate PGC-1α to drive mitochondrial biogenesis and oxidative phosphorylation [13,21,22]. Impaired lipolysis would suppress this axis, consistent with reduced PGC-1α protein observed in NLSD-M myotubes and with reduced CS activity and the hypometabolic profile observed in *PNPLA2*-mutant fibroblasts. Together, these findings highlight that both LD quantification and comprehensive metabolic profiling are necessary endpoints for drug screening in NLSD-M in vitro models.

Under fenofibrate exposure, most measured bioenergetic parameters in the patient-derived line were closer to the corresponding values in treated controls, consistent with PPARα activation partially restoring mitochondrial oxidative capacity—supporting impaired PPARα–PGC-1α signaling, rather than intrinsic ETC dysfunction, as a primary driver of the hypometabolic phenotype [22,23]. Concordantly, PPARδ agonism improved respiration by 57–63% in healthy primary myotubes but was profoundly blunted or absent in NLSD-M myotubes in proportion to ATGL deficiency severity, with reduced PGC-1α protein in both patients—consistent with functional ATGL being required for PPAR agonism to improve mitochondrial respiration, and suggesting that the bioenergetic normalization observed under fenofibrate in our fibroblast model reflects sufficient residual ATGL activity to permit a PPAR response [13]. The paradoxical co-occurrence of metabolic profile similar to that of controls and increased LD burden with fenofibrate may be explained by the dual role of PPARα activation: while enhanced FAO of exogenous fatty acids restores the bioenergetic profile, simultaneous upregulation of fatty acid uptake via CD36 and fatty acid transport proteins increases intracellular fatty acid influx; in cells with impaired ATGL-mediated lipolysis, this additional substrate cannot be efficiently mobilized from LD and is instead redirected toward de novo LD biogenesis. This hypothesis is exploratory—measuring PPARα target gene expression, fatty-acid uptake rates, and isotope-traced lipid partitioning in fenofibrate-treated *PNPLA2* fibroblasts would be necessary to confirm this mechanism. A previous study reported decreased skeletal muscle lipid content with longitudinal bezafibrate treatment in NLSD-M patients [12], contrasting with increased LD burden observed here; this discrepancy likely reflects differences between primary muscle tissue and fibroblast models, and between bezafibrate (pan-PPAR agonist) and fenofibrate (primarily PPARα agonist).

Lomitapide did not alter LD burden after two weeks of continuous treatment, contrasting with the report of reduced LD area in NLSD-I fibroblasts—a 2000-fold higher concentration over a substantially shorter treatment period (24 h vs. 2 weeks) [11], precluding direct comparison. Lomitapide inhibits microsomal triglyceride transfer protein, reducing very-low-density lipoprotein assembly and circulating lipid availability; in NLSD-M fibroblasts with pre-existing lipolytic deficiency, substrate deprivation may compound metabolic dysfunction—consistent with worsening across nearly all bioenergetic readouts and a further reduction in CS activity. Furthermore, LD–mitochondria contact sites are already reduced in NLSD-M myotubes, suggesting that substrate deprivation via lomitapide could compound a pre-existing defect in LD-to-mitochondria lipid transfer [13]. However, LD–mitochondria interactions were not assessed in this study.

Several methodological considerations are relevant. ORO was selected as a well-established neutral lipid stain compatible with fixed-cell confocal imaging and previously used in NLSD studies [3,11]. BODIPY™ 493/503 represents an alternative approach, although differences in signal-to-noise characteristics may be cell-type and protocol dependent [20,24,25]. Importantly, our comparison of imaging approaches demonstrated that single-plane analysis systematically underestimated LD number and overestimated apparent LD size relative to volumetric z-stack analysis. This methodological bias becomes particularly relevant in cells containing numerous small or densely clustered droplets and supports the preferred approach of three-dimensional quantification for therapeutic studies in which changes in LD number and morphology may be biologically distinct. Finally, LD quantification was performed across multiple fields of view within a single patient-derived cell line; as fields of view within the same well do not constitute independent biological replicates, treatment-related differences in LD parameters should be interpreted as exploratory.

This study has several limitations. Most importantly, the findings derive from a single patient cell line and therefore cannot establish whether the observed phenotype or treatment responses generalize across the genetically and clinically heterogeneous NLSD-M population. The use of multiple control lines and conservative comparison with 95% and 99% CIM strengthened interpretation of this *N*-of-1 design but does not substitute for replication in additional *PNPLA2* genotypes. Control fibroblasts were derived from donors aged 9–37 years; as mitochondrial content in primary dermal fibroblasts is known to decline with donor age [26], age-range differences between the patient (32 years), and the younger controls may contribute to the observed reduction in CS activity. Nevertheless, leave-one-out sensitivity analysis demonstrated that the reduced CS activity and the broader hypometabolic bioenergetic profile remained outside control confidence intervals regardless of which control line was excluded, suggesting these findings are not attributable to the influence of any single control line (Figure S3). However, donor-age differences and incompletely documented passage matching may contribute to inter-line variability, and leave-one-out analyses cannot fully exclude these effects. Healthy-control LD measurements were not obtained, and LD treatment comparisons were performed within a single biological culture per condition, with imaging fields providing technical sampling rather than independent biological replication. FAO was not measured directly, and PNPLA2 abundance, PPARα target-gene expression, and PGC-1α signaling were not assessed, preventing direct confirmation of the proposed ATGL–PPARα–PGC-1α mechanism. Autophagy was evaluated using static LC3 B-II/LC3 B-I abundance as an indicator of basal autophagosome accumulation; as this does not measure autophagic flux, altered flux due to compensatory changes in autophagosome formation and clearance cannot be excluded [18]. Treatment was examined at a single concentration and duration. Future studies should therefore validate these findings across additional NLSD-M cell lines and incorporate dose-response studies, direct FAO measurements, lipid-flux analysis, and molecular interrogation of PPARα signaling. Findings in dermal fibroblasts require confirmation in additional patient lines and skeletal-muscle-relevant models, as divergent mitochondrial phenotypes between cell types suggest tissue-specific differences and may influence both disease expression and therapeutic response evaluation in NLSD-M [13].

Despite these limitations, this study further illustrates how patient-derived fibroblasts can serve as a platform for personalized therapeutic evaluation in ultra-rare disorders such as NLSD-M, while recognizing that single-cell-line findings require validation. The discordance between lipid storage and bioenergetic responses demonstrates that LD burden alone may not adequately predict therapeutic benefit, supporting the combined use of quantitative LD phenotyping and metabolic profiling as complementary endpoints. In this patient, these findings informed clinical management: lomitapide was not pursued given worsened bioenergetics without LD reduction, and fenofibrate was discontinued as a precautionary decision following paradoxical LD accumulation in vitro. Although in vitro *N*-of-1 findings cannot establish efficacy or safety, and fibroblast responses may not translate directly to affected tissues, this approach demonstrates how patient-specific functional testing may complement existing evidence in therapeutic decision-making for NLSD-M and other ultra-rare metabolic disorders.

## 4. Materials and Methods

### 4.1. Case Report

The patient described here was first reported by Akman et al. (2010) [15] at age 18 as a case of NLSD-M presenting with subclinical hyperCKemia and lipid storage in the absence of weakness; the authors noted plans for in vitro fibroblast studies to evaluate pharmacological interventions. Subsequent follow-up by Missaglia et al. (2022) [27] reported disease progression to age 30 under medium-chain triglycerides oil supplementation, which reduced serum creatine kinase but did not halt myopathy progression. The present report further extends follow-up to age 32, documenting continued disease progression and reporting the in vitro pharmacological evaluation of fenofibrate and lomitapide.

The patient is now a 32-year-old female experiencing progressing muscle weakness, difficulties walking, and shortness of breath with exertion (Appendix A). First abnormal findings of elevated serum creatine kinase, transaminases, and fatty infiltration in leg muscles on MRI were found at 10–12 years of age still in asymptomatic period. Deltoid muscle biopsy (age 15 years) confirmed lipid myopathy. At the age of 17 years (reported as age 18 years in Akman et al., 2010 [15]), *PNPLA2* sequencing revealed homozygous pathogenic frameshift variant in exon 3 (*PNPLA2*:c.231_232 ins?, p.(Lys78 fs); ClinVar variation ID 2735395) and NLSD-M was diagnosed. Since the initial report, repeated skeletal muscle MRIs (ages 23, 27, and 32 years) showed progressing lipid infiltration [27]. Liver (age 31 years) and cardiac MRIs (ages 30 and 31 years) had no lipid infiltration and no signs of cardiomyopathy.

First presentation of progressive muscle weakness in upper extremities was reported at around 20 years; lower extremities weakness became apparent 5–6 years later. The latest physical examination at 32 years was remarkable for global hypotonia, bilaterally reduced strength in both upper and lower extremities, with hands more severely affected, and broad-based and waddling gait.

As of last assessment (age 32 years), patient continues physical and occupational therapy. Her current medications and supplements include: NADH, Nutri-West, vitamin D, glutathione, L- carnitine, ubiquinol, B group vitamins, triheptanoin, fenofibrate.

### 4.2. Fibroblasts Cell-Line Maintenance

NLSD-M primary skin fibroblast cultures were established from punch biopsies obtained with informed consent. Biopsy tissue was seeded onto 12.5 cm^2^ tissue culture flasks in RPMI 1640 (Gibco, Thermo Fisher Scientific, Grand Island, NE, USA) supplemented with 18% heat inactivated fetal bovine serum (Gibco, Thermo Fisher Scientific, Grand Island, NE, USA), 1% L-glutamine (Gibco, Thermo Fisher Scientific, Grand Island, NE, USA), and 1% antibiotic-antimycotic (Gibco, Thermo Fisher Scientific, Grand Island, NE, USA). Flasks were incubated at 37 °C and 5% CO_2_ to allow tissue adhesion and fibroblast outgrowth. Cultures were fed every 48–72 h and monitored for fibroblast emergence. Upon reaching 80% confluence, cells were passaged using Trypsin-EDTA and expanded for downstream experiments. Next, NLSD-M and control fibroblasts (Table S2) were maintained with Dulbecco’s Modified Eagle Medium (DMEM), high glucose, GlutaMAX^TM^ Supplement, pyruvate (Gibco, Thermo Fisher Scientific, Grand Island, NE, USA) supplemented with 10% (*v*/*v*) heat inactivated fetal bovine serum (Gibco, Thermo Fisher Scientific, Grand Island, NE, USA) and 1% (*v*/*v*) Antibiotic–Antimycotic (Gibco, Thermo Fisher Scientific, Grand Island, NE, USA), at 37 °C and 5% CO_2_. Passage numbers were recorded within the overall range of 6–14 across all lines and experiments; per-line per-experiment passage tracking was not performed, and a contribution of passage-related variability to inter-line differences cannot be formally excluded.

### 4.3. Seahorse Cellular Respirometry

Oxygen consumption and extracellular pH were assayed using the XFe96 Analyzer (Agilent Technologies, Santa Clara, CA, USA) and the XF Cell Mito Stress Test kit (Agilent Technologies, Santa Clara, CA, USA) as previously described [28,29,30]. Briefly, NLSD-M and control fibroblasts were seeded 10,000 cells/well on 96-well XF Cell Culture Plates (Agilent) and cultured for 72 h. Prior to the assay, standard culture media was replaced with XF Dulbecco’s Modified Eagle Medium (DMEM) supplemented with 10 mM XF glucose, 1 mM XF pyruvate, and 2 mM XF glutamine. Oligomycin (2.5 μM), carbonyl cyanide *p*-trifluoromethoxyphenylhydrazone (FCCP) (2 μM), a mixture of rotenone/antimycin A (0.5 µM each) were administered sequentially to each well. Oxygen concentration and extracellular pH were measured prior and after each toxin injection. Proton efflux rate (PER) was calculated by multiplying the rate of change in pH (extracellular acidification rate, ECAR) at each time point by the buffering capacity of the media and plate volume. Each sample had at least three technical replicates. Derived parameter definitions are detailed in Table S1.

All Seahorse metabolic analysis parameters were normalized to protein concentration, a proxy readout of cell mass, and citrate synthase (CS) abundance/activity, a proxy readout for mitochondrial content. Following the last respirometry measurement, cells were lysed by incubation with 10 mM Tris-HCl (Thermo Fisher Scientific Inc., Waltham, MA, USA) plus 0.3% Triton X-100 (Millipore Sigma, Burlington, MA, USA). Lysates were incubated with acetyl-CoA (Millipore Sigma, Burlington, MA, USA), oxaloacetate, and 5,5′-dithiobis-(2-nitrobenzoate) (DTNB) (Millipore Sigma, Burlington, MA, USA). CoA-SH generated by the condensation of acetyl-CoA and oxaloacetic acid by CS to citric acid rapidly cleaves DTNB (colorless) to TNB-, which rapidly ionizes to TNB2- (yellow). Condensation of acetyl-CoA and oxaloacetic acid by CS was assayed via spectrophotometric measurement of TNB2- at 411 nm over 15 min using VANTAstar (BMG LABTECH GmbH, Ortenberg, Germany) reader. Protein concentration of cell homogenates was assayed using the Pierce BCA Protein Assay Kit (MilliporeSigma, Burlington, MA, USA).

### 4.4. Mitochondrial Enzymology

Total cellular NADH oxidation capacity and Complex II (CII) activity were assayed spectrophotometrically as previously described [31]. Briefly, whole-cell lysates were prepared by bead-mill homogenization of approximately 5–10 million fibroblasts in 20 mM Tris-HCl (pH 7.4) with 1.4 mm ceramic beads (Omni International, Kennesaw, GA, USA) and assayed on a VANTAstar plate reader (BMG LABTECH GmbH). For total NADH oxidation capacity, cell homogenates were incubated in potassium phosphate (K_2_ HPO_4_)-buffered solution containing bovine serum albumin (BSA) (Millipore Sigma, Burlington, MA, USA), NADH (Roche, Basel, Switzerland), ubiquinone (CoQ) (Millipore Sigma), and the artificial electron acceptor 2,6-dichlorophenolindophenol (DCPIP) (Millipore Sigma). Reduction of ubiquinone to ubiquinol by NADH oxidation rapidly reduces DCPIP (blue) to DCPIH_2_ (colorless); total NADH oxidation capacity was assayed via spectrophotometric measurement of DCPIP absorbance extinction at 600 nm over 17 min. As rotenone was not included in this assay, the measured rate reflects total cellular NADH oxidation rather than isolated Complex I activity. For CII activity, cell homogenates were incubated in K_2_ HPO_4_-buffered solution containing BSA, EDTA (Millipore Sigma), sodium azide (Millipore Sigma), succinate (Millipore Sigma), decylubiquinone (DUB) (Millipore Sigma), ATP (Millipore Sigma), and DCPIP. Succinate oxidation by CII reduces DUB, which in turn rapidly reduces DCPIP; CII activity was assayed via spectrophotometric measurement of DCPIP absorbance at 600 nm over 15 min. Non-specific DCPIP reduction was corrected by simultaneously assaying each homogenate in the presence of the CII inhibitor malonate (Millipore Sigma), and CII activity was calculated by subtracting malonate-inhibited from total succinate oxidation. All enzyme activity assays were performed in two independent experimental runs; values were averaged across runs prior to normalization. All enzyme activities were normalized to protein concentration and CS activity as described above.

### 4.5. Lipid-Droplet Quantification

LD content was assessed by Oil Red O (ORO) staining. *PNPLA2*-mutant fibroblasts were seeded in Nunc Lab-Tek II CC2 Chamber Slides (Thermo Fisher Scientific, Grand Island, NE, USA, 154941) at ~2500 cells/chamber and cultured for 72 h. Cells were fixed in 4% paraformaldehyde for 30 min at room temperature and incubated in 60% isopropanol for 30 s. ORO working solution (3 parts 0.5% ORO in isopropanol, 2 parts ddH_2_ O, filtered through 0.22 µm) was applied for 10 min at room temperature, followed by ddH_2_ O washing. Nuclei were counterstained with DAPI using VECTASHIELD Antifade Mounting Medium (Vector Laboratories, Newark, CA, USA, H-1200-10). Images were acquired on a Zeiss LSM980 Airyscan confocal microscope (Carl Zeiss AG/Carl Zeiss Microscopy GmbH, Oberkochen, Germany). Fiji/ImageJ (version 2.16.0/1.54r) and Imaris (Oxford instruments, High Wycombe, United Kingdom, version 11.0) were used for image analysis and quantification.

LD burden was not assessed in control fibroblasts, as the established accumulation of neutral lipids in NLSD-M dermal fibroblasts relative to healthy controls is well documented [14,15,16], and the primary aim of the LD analysis was to characterize compound-specific responses within the patient-derived line rather than to recapitulate the diagnostic phenotype.

### 4.6. Western Blot

Protein was extracted from confluent fibroblast monolayers by scraping in ice-cold PBS, pelleting by centrifugation (500× *g*, 5 min), and lysing in RIPA buffer (Sigma Aldrich R0278) supplemented with protease inhibitor cocktail (Sigma Aldrich P8340, 1:100) prepared fresh on the day of extraction. Cells were homogenized by passage through a 25 G insulin syringe followed by 30 min incubation on ice with periodic vortexing and clarified by centrifugation at 14,000× *g* for 20 min at 4 °C. Protein concentration of cell homogenates were assayed using the Pierce BCA Protein Assay Kit (Millipore). Samples (30 µg) were prepared in 2× Laemmli sample buffer (Bio-Rad, Hercules, CA, USA #161-0737) with 5% 2-mercaptoethanol (BME, Sigma Aldrich, St. Louis, MO, USA M6250–100 ML), denatured at 95 °C for 5 min, resolved on 4–20% Criterion Tris-HCl gradient gels (Bio-Rad #3450032) in Tris/glycine/SDS running buffer, and transferred to 0.2 µm nitrocellulose membranes using the Trans-Blot Turbo RTA Kit (Bio-Rad #1704270). Transfer quality was confirmed by Ponceau S staining. Membranes were blocked in fish serum blocking buffer for 1 h at room temperature and incubated 24 h at 4 °C with primary antibodies against LC3 B (Novus NBP2-46892, rabbit, 1:500) and β-actin (ABclonal AC004, mouse, 1:10,000). Membranes were incubated with IRDye 800 CW anti-rabbit and IRDye 680 RD anti-mouse secondary antibodies (1:10,000) for 1 h at room temperature and imaged on a LI-COR Odyssey CLx infrared imaging system. Relative protein abundance was assessed as band intensities quantified in Fiji/ImageJ. Background correction was applied as: corrected intensity signal = RawIntDen − (Area x Mean background), where Mean background was calculated as the average of per-pixel intensities (RawIntDen/Area) from signal-free regions of interest distributed across the membrane. LC3 B-I and LC3 B-II were normalized to β-actin and LC3 B-II/LC3 B-I ratio was calculated. Technical replicates within each gel were averaged per line per gel, independent gels were treated as separate technical replicates and were averaged per line.

### 4.7. Treatment

Fenofibrate (Thermo Fisher Scientific, Grand Island, NE, USA, cat. no. 461780050) was dissolved at 200 mM and lomitapide (Selleck Chemicals, Houston, TX, USA cat. no. S7635) at 10 mM in DMSO. Culture media was supplemented with Fenofibrate 20 µM (0.01% *v*/*v* DMSO; reflecting plasma levels of 19–30 µmol/L), lomitapide 5 nM (0.01% *v*/*v* DMSO; reflecting plasma levels of 5–7 nmol/L), or vehicle (0.01% *v*/*v* DMSO) for two weeks prior to Seahorse oximetry, ORO staining, and cell harvesting. During two-week treatment, media was changed every 3–4 days with compounds or vehicle refreshed every time. Untreated conditions were matched by the same duration time in culture. Both *PNPLA2*-mutant and control cell lines were treated.

### 4.8. Statistical Analysis

All measurements represent independent biological replicates; technical replicates were averaged per cell line. To compare the single *PNPLA2* patient cell line against control cell lines, a T-score was computed independently for each parameter: T = (x_patient − $x^{¯}$_control)/(s_control/√*n*), where $x^{¯}$_control and s_control are the mean and standard deviation of the control cell lines and *n* is the number of control cell lines. Two-tailed significance thresholds were defined by the T-score at df = *n* − 1 of T-distribution. The 95% and 99% confidence intervals of the control mean (CIM) were computed as $x^{¯}$_control ± t_crit × (s_control/√*n*), where s_control is the sample standard deviation of the control cell lines and t_crit corresponds to the 97.5th and 99.5th percentiles of the t-distribution at df = *n* − 1, respectively. A T-score exceeding the 95% or the 99% CI boundary was considered to indicate a statistically meaningful deviation from the control mean. To assess whether any single control line disproportionately influenced the patient comparisons, a leave-one-out sensitivity analysis was performed in which *PNPLA2*-mutant fibroblasts were compared against each possible subset of four controls. All analyses and visualizations were performed in R using RStudio (version 2025.09.1+401).

## Data Availability

The data presented in this study are available on reasonable request from the corresponding author.

## Supplementary Materials

The following supporting information can be downloaded at: https://www.mdpi.com/article/10.3390/ijms27219609/s1.

## Abbreviations

The following abbreviations are used in this manuscript:

ATGL	Adipose Triglyceride Lipase
CII	Complex II
CIM	Confidence intervals of the control mean
CS	Citrate synthase
ECAR	Extracellular acidification rate
ETC	Electron Transport Chain
FAO	Fatty Acid Oxidation
PPAR	Peroxisome Proliferator-Activated Receptor
PGC-1 α	Peroxisome Proliferator-Activated Receptor Gamma Coactivator 1-alpha
LD	Lipid droplet
NLSD-I	Neutral lipid-storage disorder with ichthyosis
NLSD-M	Neutral lipid-storage disorder with myopathy
OCR	Oxygen-consumption rate
ORO	Oil Red O
OxPhos	Oxidative phosphorylation
PER	Proton efflux rate
SE	Standard error

## Appendix A. NLSD-M Case Report

A 32-year-old female (full-time school psychologist) experiences progressing muscle weakness, difficulties walking, and shortness of breath with exertion.

Pregnancy, birth, and early childhood were uneventful, no developmental issues, no milestone regression. At 10 years patient was evaluated for possible viral infection; further workup revealed elevated serum creatine kinase, transaminases, and MRI (age 12 years) findings indicative of fatty infiltration of the leg muscles with sparing of the abductors that led to inflammatory myopathy diagnosis. Deltoid muscle biopsy (age 15 years) confirmed lipid myopathy. Lipid accumulation was seen on a tonsillectomy, wound debridement, and in peripheral blood as Jordan’s anomaly [21]. At the age of 17 years, PNPLA2 sequencing revealed homozygous pathogenic frameshift variant PNPLA2:c.231_232 ins?, p.(Lys78 fs) and NLSD-M was diagnosed.

Patient was a competitive dancer ages 3–20 years without muscle-weakness complaints. At the age of 20 years old, she developed right shoulder weakness that slowly progressed distally over two years. Physical and occupational therapy eventually plateaued without improvement ending her dancing career aged 21 years. At ages 24–25 years, she developed similar left-shoulder weakness that progressed distally. First lower extremities symptoms were noted around 26 years with difficulties walking, which progressed ever since. Worsening muscle weakness negatively impacted her everyday life, e.g., difficulties lifting small objects like glass of water, putting on clothes by herself. Patient has no muscle fatigability, no difficulty speaking, chewing, breathing, or swallowing, and no problems holding her neck up. Latest physical examination at 32 years was remarkable for global hypotonia, bilaterally reduced strength in both upper and lower extremities, with hands more severely affected. Patient had broad-based, waddling gait. Patient’s gait abnormalities were more prominent in the dark or with eyes closed. Patient uses walker to ambulate independently in unfamiliar environments.

Repeated skeletal muscle MRIs (ages 23, 27, and 32 years) showed progressing fat infiltration: from tibialis anterior and gastrocnemius muscles progressing towards high-grade symmetrical fatty infiltration of the bilateral thighs and pelvis (bilateral gluteus and almost all the posterior compartment musculature and vastus muscles) and fat-tissue replacement of deltoid and biceps muscles in the right upper extremity. Liver MRI (age 31 years) showed no lipid infiltration and repeated cardiac MRIs (ages 30 and 31) had no lipid infiltration and preserved normal geometry and function.
