# Supplementary material for: Fenofibrate and Lomitapide in PNPLA2-Related Neutral Lipid-Storage Disease with Myopathy: A Single-Patient In Vitro Approach
Source: Int J Mol Sci. 2026 Oct 27;27(21):9609. doi: 10.3390/ijms27219609 (PMC13763481; doi:10.3390/ijms27219609)
Supplement: Supplementary file 1 [file ijms-27-09609-s001.zip › ijms-4547693-supplementary.pdf]

**Table S1.** Seahorse XF Mito Stress Test parameter definitions.

| Parameter | Definition | Calculation |
| --- | --- | --- |
| Non-mitochondrial OCR | Oxygen consumption rate (OCR, pmol $O_2$/min) from non-mitochondrial sources. | Minimum OCR following rotenone and antimycin A administration. |
| Resting OCR | Total oxygen consumption at resting conditions. | Total OCR at the final time point before oligomycin administration. |
| Basal OCR | Mitochondrial oxygen consumption at resting conditions. | Resting OCR minus non-mitochondrial OCR. |
| Mitochondrial OCR (%) | Resting mitochondrial oxygen consumption as a percentage of total resting oxygen consumption. | Basal OCR / resting OCR. |
| ATP-associated OCR | Oxygen consumption associated with ATP synthase. | OCR at the final time point before oligomycin administration, minus the minimum OCR following oligomycin administration. |
| Proton leak-associated OCR | Mitochondrial oxygen consumption following oligomycin administration. | Minimum OCR following oligomycin administration minus non-mitochondrial OCR. |
| Coupling efficiency | Percentage of basal mitochondrial OCR directed toward ATP production. | ATP-associated OCR / basal OCR. |
| Maximal OCR | Maximum OCR of the uncoupled mitochondrial respiratory chain (MRC). | Maximum OCR following FCCP administration minus non-mitochondrial OCR. |
| Spare OCR | Difference between maximal OCR of the MRC and resting mitochondrial OCR. | Maximal OCR minus basal OCR. |
| Spare OCR (%) | Spare OCR as a percentage of basal OCR. | Spare OCR / basal OCR. |
| Resting PER | Proton efflux rate (pmol $H^+$/min) under resting conditions. | PER at the last measurement before oligomycin administration. |
| Spare PER | Difference between resting and maximal glycolytic flux. | Maximal PER following oligomycin administration minus PER at the last measurement before oligomycin administration. |
| Spare PER (%) | Spare PER as a percentage of resting PER. | Spare PER / resting PER. |
| ATP (Glycolysis) | Estimated ATP production rate (pmol ATP/min) from glycolysis, derived from the proton efflux rate assuming a 1:1 relationship between glycolytic proton efflux and ATP production. | Equal to the resting PER. |
| ATP (OxPhos) | Estimated ATP production rate from mitochondrial oxidative phosphorylation, derived from ATP-associated OCR assuming a mitochondrial P/O ratio of ~2.75 (~5 ATP per $O_2$). | ATP-associated OCR x 5 (based on P/O ratio of ~2.75) |
| ATP (Total) | Estimated total ATP production rate, combining glycolytic and oxidative phosphorylation contributions. | ATP (Glycolysis) + ATP (OxPhos). |

| | | |
|---|---|---|
| ATP (OxPhos) (%) | Estimated ATP production rate from oxidative phosphorylation as a percentage of estimated total ATP production rate. | ATP (OxPhos) / ATP (Total). |

Table S1. Definitions and calculations for all Seahorse XF Mito Stress Test-derived parameters reported in this study. Estimated ATP production rates are calculated from measured oxygen consumption and proton efflux rates using established stoichiometric assumptions. OCR, oxygen consumption rate (pmol $O_2$/min); PER, proton efflux rate (pmol $H^+$/min); FCCP, carbonyl cyanide p-trifluoromethoxyphenylhydrazone; MRC, mitochondrial respiratory chain; P/O, ATP produced per oxygen atom consumed.

**Table S2. Control fibroblast cell lines.**

| ID | Sex | Age at biopsy |
|---|---|---|
| GM08399 | Female | 19 years |
| GM01948 | Female | 27 years |
| GM08400 | Female | 37 years |
| GM00038 | Female | 9 years |
| GM01651 | Female | 13 years |

Table S2. Healthy control primary skin fibroblast cell lines acquired from the Coriell Institute for Medical Research.

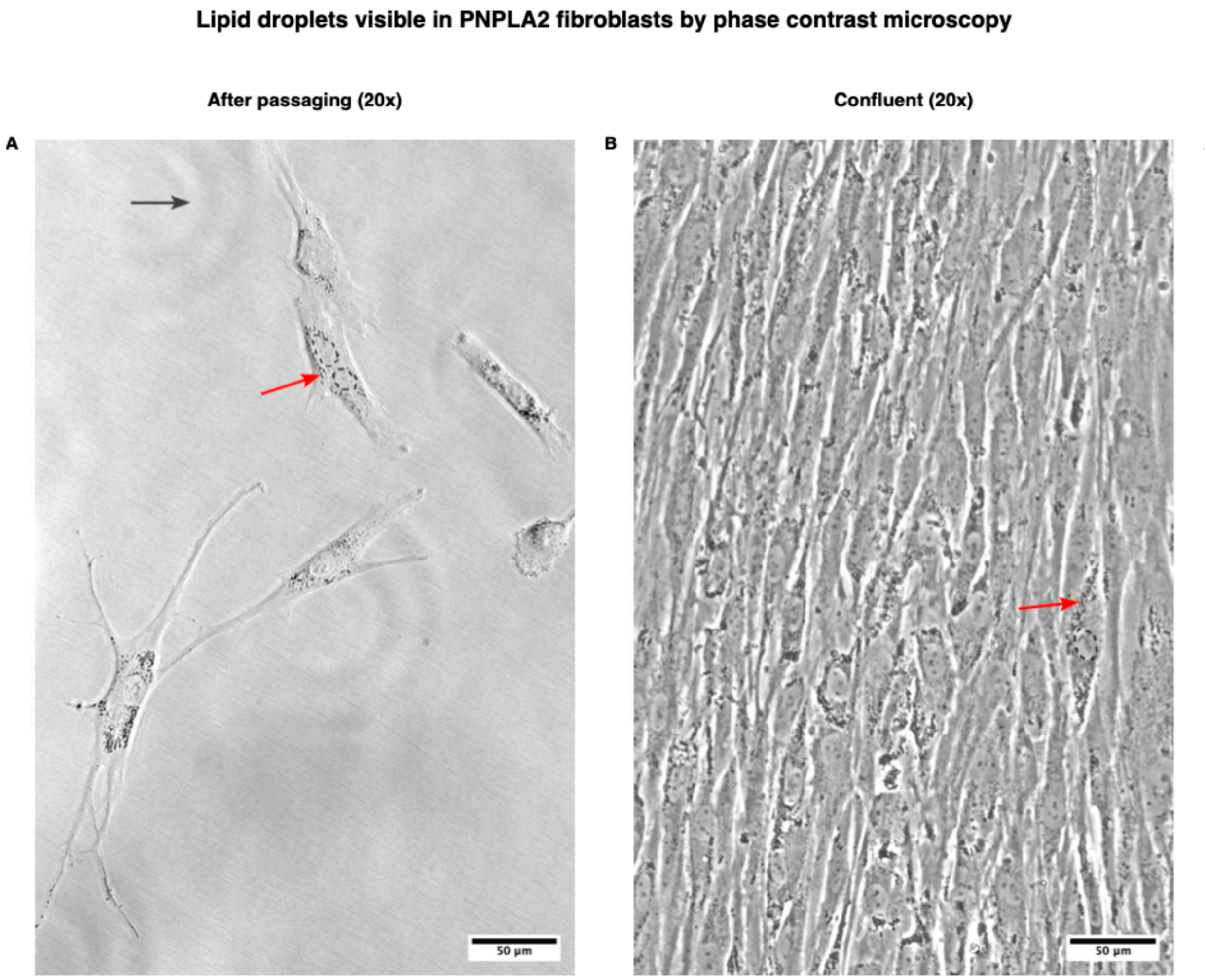


**Figure S1.** Lipid droplets in PNPLA2-mutant fibroblasts visualized by phase contrast microscopy. Representative phase contrast images of PNPLA2-mutant fibroblasts (A) shortly after passaging and (B) at confluence (20x magnification). Red arrows indicate representative lipid droplets; black arrows indicate liquid-light artefacts; dashed circles indicate estimated nuclear borders. Scale bars, 50 µm.

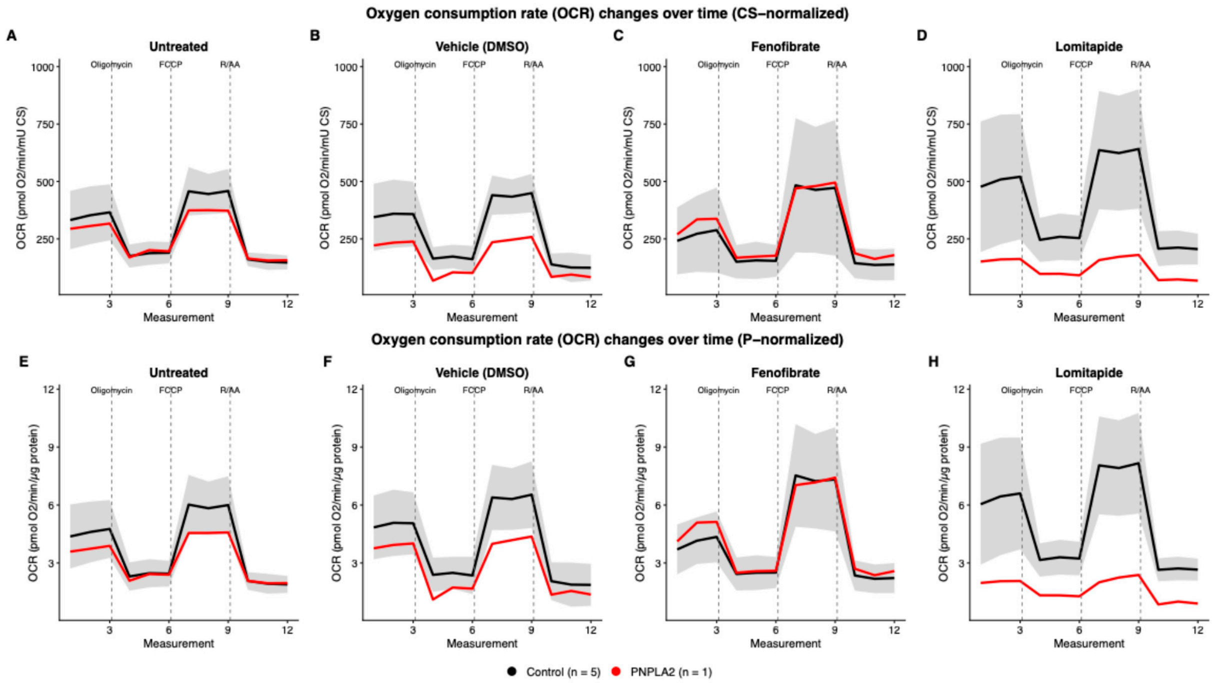


**Figure S2.** Oxygen consumption rate kinetics of PNPLA2-mutant fibroblasts and controls across treatment conditions. Oxygen consumption rate (OCR) was measured across 12 time points during the Mito Stress Test under four treatment conditions: untreated (A, E), vehicle 0.01% DMSO (B, F), fenofibrate 20 µM (C, G), and lomitapide 5 nM (D, H). Dashed vertical lines indicate sequential injections of oligomycin, FCCP, and rotenone/antimycin A (R/AA). Black line represents the mean OCR of control cell lines (n = 5); grey ribbon represents ±1 SD across controls. Red line represents the PNPLA2-mutant cell line (n = 1). Data are normalized to citrate synthase (CS) activity (A–D) and total protein content (E–H).

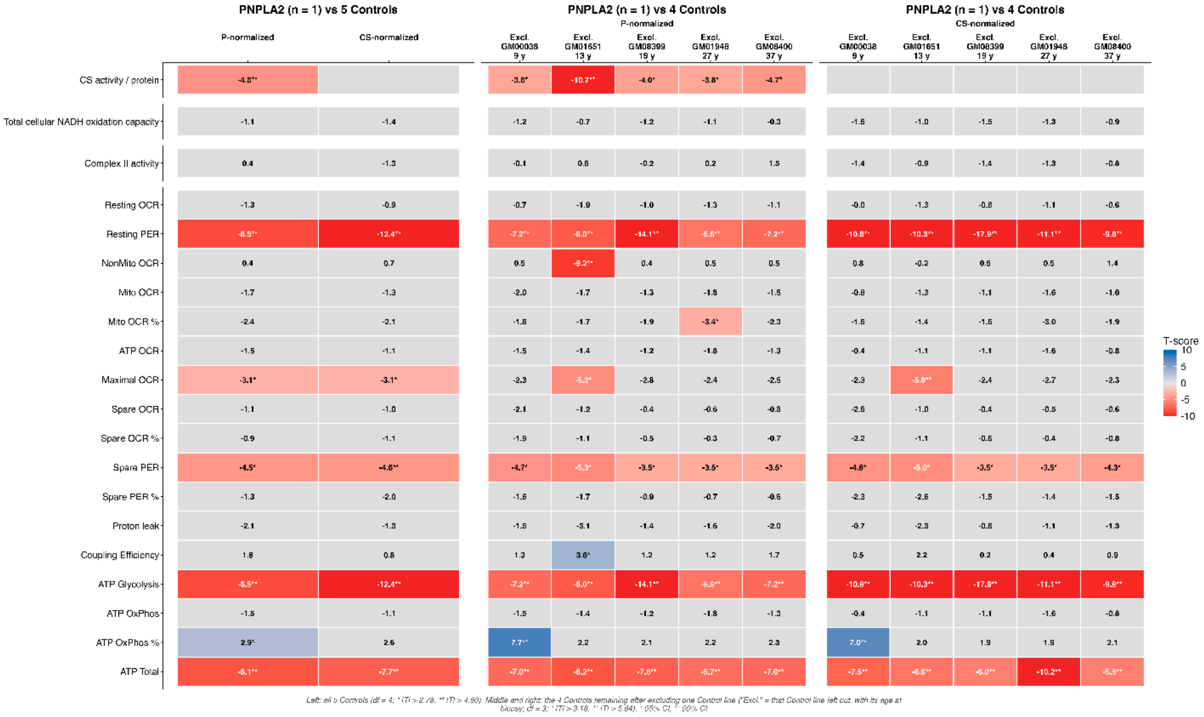


**Figure S3.** Leave-one-out sensitivity analysis of T-score comparisons between PNPLA2-mutant and control fibroblasts. Heatmaps display T-scores comparing the single PNPLA2-mutant fibroblast line to control means across Seahorse-derived bioenergetic parameters (rows), shown for protein (P)-normalized and citrate synthase (CS)-normalized data. Tile color indicates T-score direction and magnitude (blue = above controls, red = below controls); grey tiles indicate values within the 95% confidence interval of the control mean (CIM). Asterisks indicate significance thresholds: * = |T| > t-crit$_{95}$ (95% CIM); ** = |T| > t-crit$_{99}$ (99% CIM). Left panel: Full cohort comparison (n = 5 controls; df = 4; t-crit$_{95}$ = 2.78, t-crit$_{99}$ = 4.60). Middle and right panels: Leave-one-out analysis in which the patient is compared against each subset of four controls formed by excluding one control line at a time; the excluded control is identified by its line ID and donor age (years). Middle panels show P-normalized data; right panels show CS-normalized data. df = 3; t-crit$_{95}$ = 3.18; t-crit$_{99}$ = 5.84.

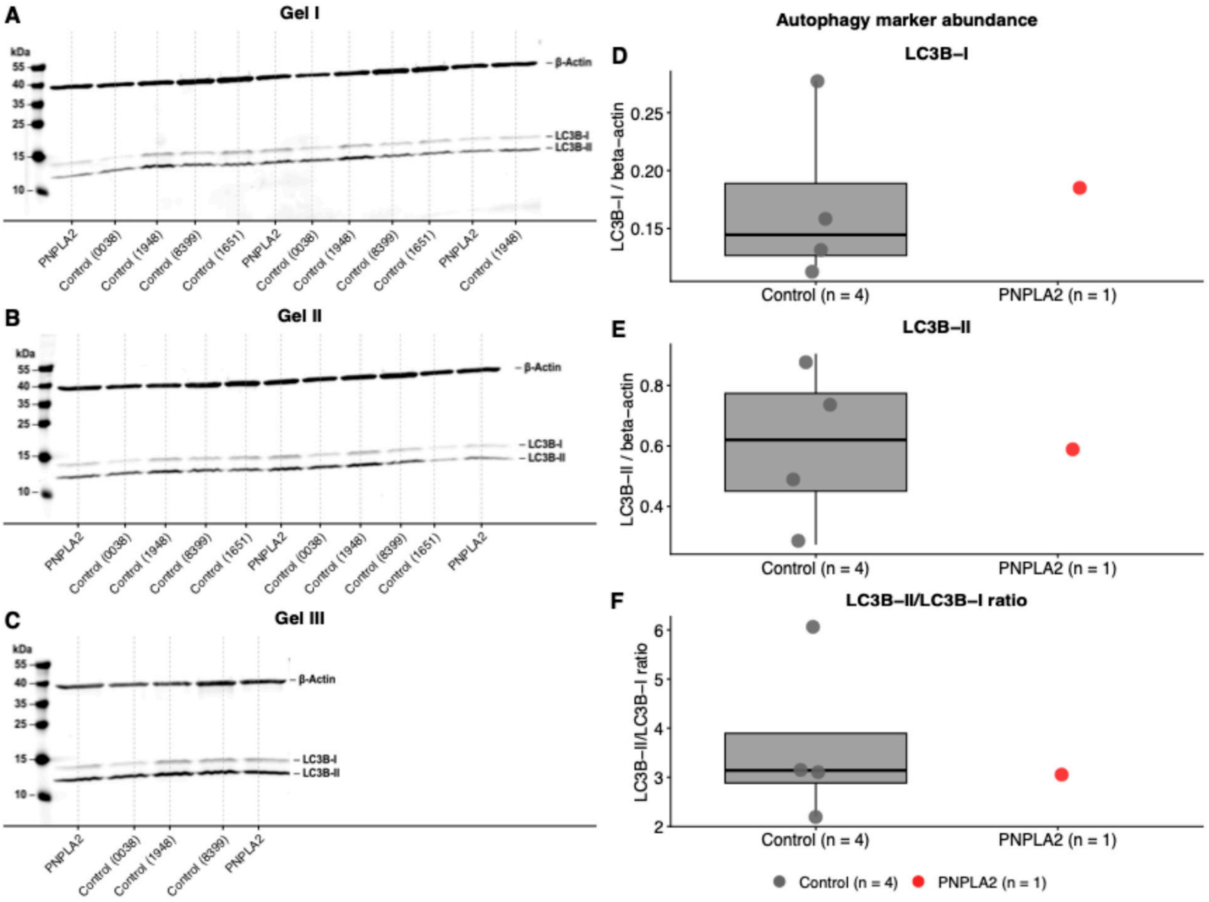


**Figure S4.** LC3B protein abundance in PNPLA2-mutant fibroblasts compared to controls. (A–C) Representative western blots (Gel I–III) probed for LC3B-I, LC3B-II, and β-actin in the PNPLA2-mutant cell line and four control cell lines (GM08399, GM01948, GM00038, GM01651). Control line GM08400 was not included in the LC3B western blot analysis as sufficient passage numbers were not available at the time these experiments were conducted. (D–F) Quantification of LC3B-I (D) and LC3B-II (E) abundance normalized to β-actin, and LC3B-II/LC3B-I ratio (F), in control cell lines (grey, n = 4) and the PNPLA2-mutant cell line (red, n = 1). Box plots show median and interquartile range of controls; individual points represent independent gel measurements. T-scores for all LC3B parameters were below the 95% CIM threshold.

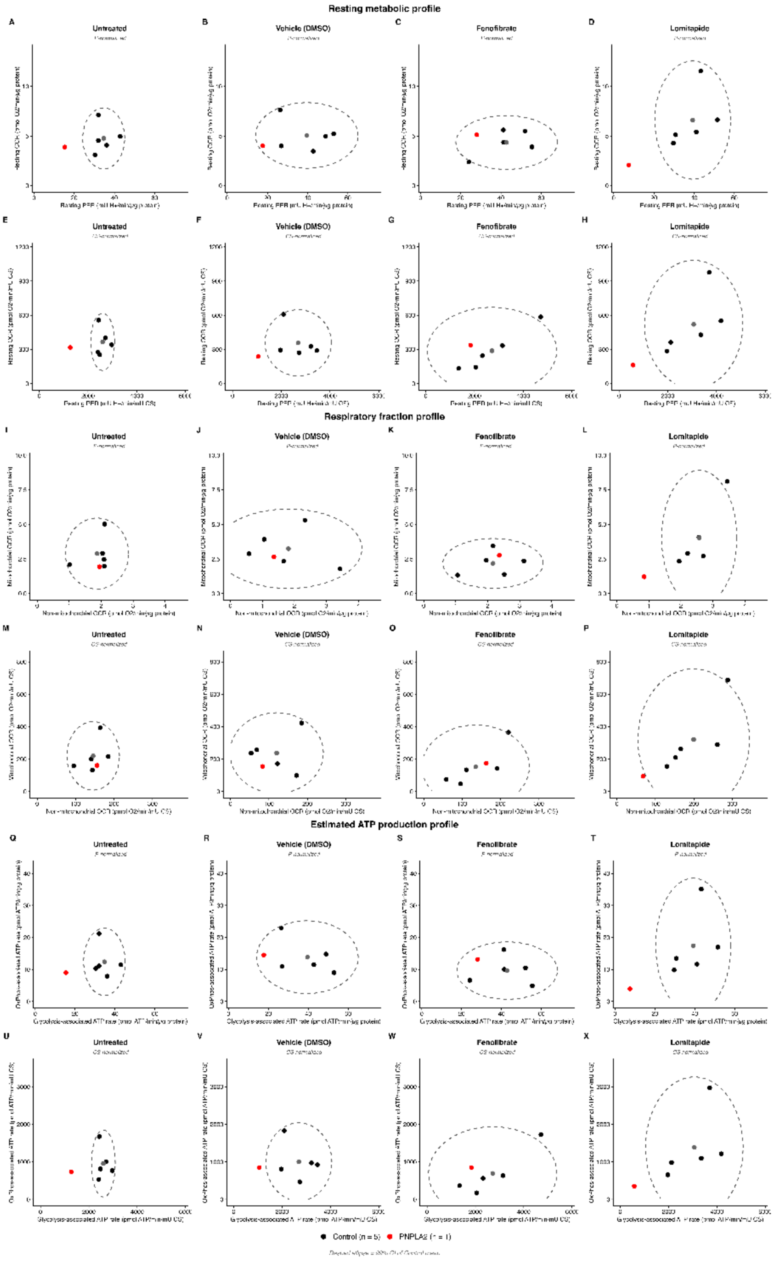


**Figure S5.** Resting metabolic, respiratory fraction, and estimated ATP production profiles of *PNPLA2*-mutant fibroblasts across treatment conditions. PNPLA2-mutant cell line (red, n = 1) and control cell lines (black, n = 5) are shown under four treatment conditions – untreated, vehicle (0.01% DMSO), fenofibrate 20 µM, and lomitapide 5 nM – with protein (P)-normalized data in the top row and citrate synthase (CS)-normalized data in the bottom row of each block. (A–H) Resting metabolic profile: resting proton efflux rate (PER, x-axis) versus resting oxygen consumption rate (OCR, y-axis). (I–P) Respiratory fraction profile: non-mitochondrial OCR (x-axis) versus mitochondrial OCR (y-axis). (Q–X) Estimated ATP production profile: PER-estimated glycolytic ATP production rate (x-axis) versus oxidative phosphorylation (OxPhos) OCR-derived ATP production rate (y-axis). Dashed ellipse indicates 99% CIM of the control group mean (grey point) within each treatment condition.
